# Variation of Ultrasonic Renal Volume between Hypertensive and Non-Hypertensive Individuals in Relation to Body Size Parameters

**DOI:** 10.4314/ejhs.v31i4.21

**Published:** 2021-07

**Authors:** Mesfin Zewdu, Elias Kadir, Melkamu Berhane, Tilehun Alemayehu

**Affiliations:** Jimma University; 1 Medical Physicist, Jimma University Radiology Department; 3 Pediatrician, Jimma University Pediatrics & Child health Department; 4 Assistant Professor, Human Anatomy, Jimma University, Department of Biomedical Sciences

**Keywords:** Ultrasonography, renal volume, hypertension, BMI, BSA

## Abstract

**Background:**

Estimation of renal size is vital for the diagnosis of abnormal structural change on the kidneys due to the adverse effects of chronic diseases like hypertension. This study evaluated renal volume by ultrasound in relation to body size parameters, notably body mass index (BMI) and body surface area(BSA) in hypertensive and non-hypertensive individuals.

**Methods:**

A hospital-based comparative cross-sectional study was conducted from February to September 2018 at the Radiology department of the Jimma University Medical Center (JUMC). The study included consecutively selected samples of 85 ambulatory hypertensive patients and 60non-hypertensive controls recruited consecutively on voluntary basis. After receiving verbal consent, each subject underwent abdominal ultrasound examination and length, width and thickness of both kidneys were measured and used for estimation of renal volume. The statistical evaluation included independent samples t-tests for mean differences with regard to ultrasonic renal measurements between hypertensive and non-hypertensive groups.

**Results:**

A total of 145 adults aged 16 – 80 years (mean ±SD=44 ±17) participated in the study. In the hypertensive group, mean renal volume of 97.7cm^3^ for the right kidney and104.4cm^3^ for the left kidney whereas in the control group, it was 101.1cm^3^ for the right and 111.8 cm^3^ for the left kidney. The mean right renal volume to BSA ratio was 58.2cm^3^/m^2^ in hypertensive group, while it was 62.6cm^3^/m^2^ among the control group (p=0.076). The mean left renal volume to BSA of the patients was 62.2cm^3^/m^2^ and significantly (p=0.012) lower than that of the non-hypertensive group, which was 69.3 cm^3^/m^2^.

**Conclusion:**

Slightly smaller bilateral renal volume among hypertensive patients as compared the controls was obtained.

## Introduction

Hypertension, defined as persistently elevated blood pressure (BP), is a multifactorial non-communicable disorder that substantially contributes to the global burden of diseases. Hypertension is a well-known modifiable risk factor for several illnesses including renal failure (1), cardiovascular diseases (2) and premature death worldwide (3). In the recent past, the prevalence and absolute burden of hypertension has raised globally, especially in low- and middle-income countries, including Ethiopia (4, 5).

The kidneys are among the organs commonly affected by hypertension (6), and hence critical targets of hypertension-induced organ damage. Understanding the early stages of the interaction between blood weight and renal work is basically vital for the essential avoidance of hypertension and renal malady. A distant better understanding of the impacts of basic hypertension on renal work may offer assistance for early location of the illness, follow-up, and to prompt treatment on evidence base (7).

Estimation of renal size could be a crucial step in the evaluation and treatment of renal illnesses (8). Renal size estimation most commonly incorporates renal length, volume and cortical thickness (9). For ordinary hone, renal length estimation is more solid because of its simple reproducibility, but most precise is the renal volume estimation (10). Additionally, the foremost exact estimation of renal state is the whole renal volume, which is related with height, weight, and adds up to body surface area (BSA) (11). In clinical practice, BSA approximates total surface area of the body and is used to calculate drug dosages and as an indicator of the health status of individuals (12).

Demonstrative imaging modalities and strategies such as computed radiography (CR), computed tomography (CT), Magnetic resonance imaging (MRI), Nuclear medicine (NM), and ultrasonography among others have been utilized for renal assessment, particularly in terms of estimate and work, but no single strategy is generally acknowledged for renal estimate appraisal (13,14,15). Even if different imaging modalities are available to be used for renal volume assessment, ultrasonography (US) has replaced standard radiography and has become the standard imaging modality in the investigation of renal diseases due to its noninvasive nature and easy availability (16). Additionally, it offers excellent anatomical details, doesn't require special patient preparation and does not expose patients to radiation or contrast agents.

Different studies have shown that anthropometric estimations like height, weight, and body mass index (BMI) relate exceptionally well with renal length and volume (17, 18). Higher BMI is associated with increased risk of several non-communicable diseases like, diabetes mellitus and hypertension, which if not treated timely and properly can lead to end-stage renal disease (ESRD) (19).

Kidney size measurements have traditionally been taken and used as predictors of chronic kidney diseases; however, these predictions are often based on an incomplete knowledge of accuracy and evolving evidence of effectiveness. Kidney length may not be an absolute predictor of overall kidney size, perhaps due in part to the fact that it measures only a single renal dimension, which is subject to inconsistency pertaining considerably to the varied shape of the kidneys within or between individuals. Renal volume (RV) rather, has been emphasized by several authors as a true predictor of kidney size in states of good health and disease (20,21).

There is no study done in Ethiopia on renal size measurements as determinant parameters either in healthy people or in those with conditions such as hypertension, diabetes mellitus, and renal disease. Therefore, this study was done with the objectives of evaluating renal volume in patients with hypertension who have not developed the chronic renal disease and correlate it with anthropometric parameters as compared to non-hypertensive controls.

## Materials and Methods

**Study area, design and subjects**: A hospital-based comparative cross-sectional study was conducted from February to September 2018 at the Radiology department of the Jimma University Medical Center (JUMC). The study participants were consecutively selected samples of hypertensive patients and non-hypertensive controls. The cases were patients with hypertension who have been on follow up at JUMC chronic illnesses follow up clinic, whereas the controls were apparently healthy individuals enrolled from the staff of the medical center and individuals of the open going by the clinic that has no known kidney issue, hypertension or diabetes. After they have been informed and their verbal consent has been received, each subject underwent abdominal ultrasound of both kidneys. During the study period, a total of 145(85 for hypertensive and 60 non-hypertensive control) underwent abdomen ultrasound examination.

**Participants' inclusion and exclusion criteria**: The hypertensive group comprised sample of adult outpatients on regular follow-up for one year or longer for established hypertension without known renal disease. Likewise, the controls were sample of similar population but without history of known hypertension or diabetes. People with chronic renal disease, pregnant women and women who had given birth in the last 12 months were excluded. On renal ultrasound, presence of two bilateral, grossly symmetric kidneys verified fulfillment of inclusion criteria of the subject (22). Further, subjects with ultrasonic evidences of abnormal kidneys such as horseshoe shaped or ectopic kidney and/or those with renal cysts were also excluded from final analysis.

**Ultrasonic examination and somatic measurements**: Participants in both study groups underwent abdominal ultrasound (US) examination with the same US machine [General Electric Health care LOGIQ P6, B-Model) using the 4 MHz curvilinear probe. Each subject had scanning of both kidneys in supine and decubitus positions in the longitudinal and transverse planes for renal length, width and antero-posterior (AP) diameter (thickness) in centimeters. The liver and spleen were used as acoustic windows for the right and left kidneys respectively (23). No prior preparations of study subjects were required before examination. Renal length (RL) was taken on a coronal scan as the longest distance between the superior and inferior poles of the kidney using an electronic caliper. The AP diameter (thickness) was also measured on the same scan as the maximum distance between the anterior and posterior walls at the mid-third of the organ. The renal width (W) was measured on a transverse scan as the longest distance between the medial and lateral borders away from the hilum of the kidney. These three measurements were later used to estimate overall renal volume (RV) of the ipsilateral kidney.

Participants were first interviewed for completed age, sex and duration of hypertension in years since diagnosis and history of kidney problems. The height (H) in meters and weight (W) in kilograms of the subjects were measured while standing erect against a ZT WHO weighing scale, and used for body mass index (BMI) and body surface area (BSA) calculations.

**Outcome measures**: The main outcome variable in this study was bilateral renal volume (RV), which was derived from the three absolute ultrasonic renal dimensions measured. On each side, renal volume was computed electronically on statistical software using an ellipsoid formula RV= RL × W × AP × 0.523 as originally described by Hricak and Lieto; 1983(24). Other variables include BMI and BSA, both derived from body weight (W) and height (H). BMI was estimated as a ratio of weight in kg to height in meter squared. Body surface area was computed using the Mosteller formula, that takes the square root of the height (m) multiplied by the weight (kg) divided by 36 (25). To account for general body physique variation among individuals with respect to renal size, renal volume to surface area ratio (RV/BSA) was also computed arithmetically as additional study variable.

**Data processing and analysis**: Collected data were checked for completeness and error, then coded and entered into Statistical Package for Social Sciences (SPSS) for windows version 23 IBM-Corp, 2015(26). Preliminary inspection of the numerical data included minimum, maximum, mean, standard deviation (SD), median and interquartile range (IQR). The statistical evaluation included independent samples t-tests for mean differences with regard to age, somatic and ultrasonic renal measurements between hypertensive and non-hypertensive groups, as well as between male and female subjects. The renal sizes on the two sides of the body were also compared with pair-sample t-tests. Bivariate correlations of the renal volume with age, body weight, height, BMI and BSA were assessed using Pearson's Product correlation coefficient (*r*), separately for the two study groups. All statistical tests were two-tailed and considered significant at *p*<0.05.

**Ethical approval**: Ethical approval was obtained from the Ethical Review Board of Jimma University, Institute of Health. Formal permission was also sought from the hospital administration and radiology department. Before enrolment, participants were informed about the study purpose and requested for their interest to participate in the study. Those who agreed and provided voluntary verbal consent were included in the study.

## Results

**Main characteristics of study participants**: A total of 145 adults (74 males and 71 females) participated in the study. They comprised 85 hypertensive outpatients (40 males and 45 female), and 60(34 male and 26 female) non-hypertensive controls. Self-reported duration of hypertension since diagnosis ranged from 1 to 24 completed years, with a mean duration of 7. The age of participants ranged from 16 – 80 with a mean (±SD) of 44 (±17) years. The mean BMI and BSA were 22.3 kg/m^2^ (range: 14.4 – 37.3) and 1.65 m^2^(range: 1.25 – 2.09) respectively (Table 1). With regard to renal size, the RRV ranged from 36.1 – 201.6 cm^3^ (mean=99.1), while LRV ranged 35.8 – 253.7 cm^3^ (mean=107.4). The RRV/BSA ranged 24.53 – 100.7(mean=60.0)cm^3^/m^2^, while LRV/BSA ranged 23.5 – 132.5 (mean=65.1)cm^3^/m^2^. Both renal volume parameters were significantly different (p<0.01) between the right and left kidneys, the left kidney being larger than the right (Table 1).

**Table 1:** Main characteristics of study participants, Jimma University Medical Center (JUMC), Jimma, Southwest Ethiopia, 2018

Variables (Valid N= 145)	Mean	SD	Minimum	Q1, 25%	Q2, Median	Q3, 75%	Maximum
Age (year)	44.4	17.3	16.0	28.0	46.0	58,0	80.0
Body weight (kg)	59.96	12.0	37.3	50.9	68.1	76,0	96.0
Body height (m)	1.640*	0.092	1.44	1.440	1.850	1.700	1.850
BMI (kg/m^2^)	22.32*	4.30	14.39	21.50	22.00	24.93	37.32
BSA (m^2^)	1.646*	0.184	1.247	1.508	1.635	1.768	2.091
Right renal length (cm)	9.598	0.957	6.970	8.035	9.570	10.375	11.890
Left renal length (cm)	9.570	0.892	6.950	9.100	9.550	10.070	12.030
Right renal width (cm)	4.956*	0.595	3.380	4.515	5.00	5.390	6.300
Left renal width (cm)	3.956	0.652	1.630	4.485	4.970	5.390	7.200
Right renal thickness (cm)	3.908a	0.540	2.700	3.460	3.900	4.200	5.700
Left renal thickness (cm)	4.242a	0.578	2820	2.830	4.200	4.620	6.180
Right renal volume (cm^3^)	99.115^b^*	28.160	36.073	80.499	96.984	118.577	201.572
Left renal volume (cm^3^)	107.416^b^	31.410	35.828	88.456	106.530	128.057	253.681
RRV/BSA (cm^3^/m^2^)	60.008^c^	14.678	24.525	50.116	58.908	68.359	100.639
LRV/BSA (cm^3^/m^2^)	65.130^c^	16.967	23.522	53.755	65.812	74.307	132.481

a,b,c: The mean values in the row are statistically significant for the right and left kidneys; BMI, body mass index; BSA, body surface area; Q1, first quartile; Q2, second quartile, Q3, third quartile; SD, standard deviation; LRV, left renal volume; RRV, right renal volume

* the mean scores are significantly different between male and female.

**Comparison of hypertensive and non-hypertensive groups**: Table 2 shows comparison of the two study groups disaggregated by sex with regard to their renal size and other variables. The mean age of the non-hypertensive group was 33 (range: 16–80) years, while that of the hypertensive was 53 (range: 20–78) years with no age difference between male and female subjects in both groups. Overall, the mean BMI was significantly higher in hypertensive group (mean= 23.4 kg/m^2^) than non-hypertensive group (20.9 kg/m^2^) in both sexes (Table 2).

**Table 2:** Comparison of ultrasonic renal volume and somatic variables between hypertensive patients and non-hypertensive controls stratified by sex, Southwest Ethiopia 2018

Variable		Non-hypertensive controls (Valid N=60)					Hypertensive patients (Valid N=85)						
	
	Sex	Valid N	Min	Max	Mean	SD	Valid N	Min	Max	Mean	SD	t-statistic	p-value
Age (year)	F	26	16.0	80.0	33.19	16.46	45	22.0	70.0	50.1	12.52	-4.878**	0.000
	M	34	18.0	63.0	32.23	13.25	40	20.0	78.0	55.75	15.06	-7.070**	0.000
	F + M	60	16.0	80.0	32.65	14.60	85	20.0	78.0	52.76	13.98	-8.377**	0.000
Body weight (kg)	F	26	37.3	80.0	56.71	11.37	45	41.0	96.0	61.70	14.47	-1.507	0.136
	M	34	43.8	77.3	57.20	7.66	40	45.0	86.0	62.46	11.86	-2.297	0.025
	F + M	60	37.3	80.0	56.99	9.36	85	41.0	96.0	62.06	13.24	-2.701*	0.008
Body height (m)	F	26	1.44	1.76	1.58^a^	0.07	45	1.47	1.80	1.58^c^	0.06	-0.019	0.985
	M	34	1.52	1,85	1.72^a^	0.07	40	1.50	1.82	1.69^c^	0.07	1.746	0.085
	F + M	60	1.44	1.85	1.66	0.10	85	1.47	1.82	1.63	0.08	1.709	0.090
BMI (kg/m^2^)	F	26	14.39	29.38	22.70^b^	3.72	45	19.0	37.32	24.65^d^	4.93	-1.750	0.084
	M	34	15.47	25.46	19.46^b^	2.53	40	16.56	32.39	21.88^d^	3.59	-3.389*	0.001
	F + M	60	14.39	29.38	20.86	3.47	85	16.56	37.32	23.35	4.54	-3.562**	0.000
BSA (m^2^)	F	26	1.23	1.92	1.57	0.18	45	1.32	2.09	1.64	0.21	-1.316	0.192
	M	34	1.41	1.94	1.65	0.12	40	1.42	2.01	1.71	0.18	-1.597	0.115
	F + M	60	1.25	1.94	1.61	0.15	85	1.32	2.09	1.67	0.20	-1.829	0.069
RRV (cm^3^)	F	26	61.75	159.5	96.41	24.53	45	45.81	163.9	92.63	23.52	0.643	0.522
	M	34	63.72	152.7	104.68	22.65	40	36.07	201.6	103.44	36.68	0.176	0.861
	F + M	60	61.75	159.5	101.10	24.20	85	36.07	201.6	97.72	30.72	0.711	0.478
LRV (cm^3^)	F	26	35.83	253.7	105.21	40.67	45	53.71	162.7	102.9	25.64	0.294	0.770
	M	34	77.06	169.1	116.76	22.40	40	39.56	189.5	105.98	36.20	1.556	0.122
	F + M	60	35.83	253.7	111.76	31.86	85	39.56	189.5	104.35	30.91	1.405	0.162
RRV/BSA (cm^3^/m^2^)	F	26	41.9	96.1	61.48	14.33	45	33.62	99.1	56.58	12.31	1.520	0.133
	M	34	37.0	93.4	63.43	13.01	40	23.53	100.6	60.01	18.01	0.924	0.358
	F + M	60	37.0	96.1	62.58	13.51	85	23.53	100.6	58.19	15.26	1.789	0.076
LRV/BSA (cm^3^/m^2^)	F	26	23.52	132.5	67.10	22.85	45	38.6	97.4	63.04	14.64	0.905	0.369
	M	34	41.5	104.1	71.01	13.10	40	24.05	94.7	61.23	16.99	2.734*	0.008
	F + M	60	23.62	132.5	69.30	17.92	85	24.05	97.4	62.19	15.71	2.532*	0.012

F, female; M, male; BMI, body mass index; BSA, body surface area; Max, maximum; Min, minimum; SD, standard deviation; ^a,b,c,d,e^, the values indicated are statistically significant for the right and left kidneys; LRV, left renal volume; RRV, right renal kidney volume

* significant at p<0.01

** significant at p<0.001 between men and women

In the hypertensive group, renal volume of both sexes ranged from 36.1 to 201.6(mean=97.7) cm^3^ for the right kidney and 39.6 to 189.5 (mean=104.4) cm^3^ for the left kidney. In this group, mean volumes of the right and left kidneys in males were 103.4(±36.7) and 106.0 (±36.2) cm^3^respectively, while it was 92.6(±23.5) and 102.9 (±25.6) cm^3^ respectively for females (Table 2). In the non-hypertensive group, the renal volume ranged from 61.8–159.5(mean=101.1) cm^3^ for the right and 35.8 – 253.7 (mean=111.8) cm^3^ for the left kidney, indicating slightly larger kidneys on both sides in this group as compared to the hypertensive group.

When renal volume on each side is seen in terms of body surface area, RRV/BSA ranged from 23.5–100.6(mean=58.2) cm^3^/m^2^ in the hypertensive group, while it was between 37.0 – 96.1(mean=62.6) cm^3^/m^2^ among the nonhypertensive group (p=0.076). In contrary, LRV/BSA of the hypertensive group ranging from 24.1 – 97.1 (mean=62.2) cm^3^/m^2^ was significantly (p=0.012) lower than that of the non-hypertensive group, which was 23.6–132.5 (mean=69.3) cm^3^/m^2^ (Table 2).

**Factors associated with renal volume in the study population**: Relationship of the RRV and LRV with age, weight, height, BMI and BSA was shown in Table 3. As shown, neither the right nor the left renal volume has significant correlation with age in either group or sex. The largest mean renal volumes for right and left kidney were recorded in same age group 40–49 years in the male and female hypertensive subjects, in the control group however largest renal volumes were calculated for those in the fourth decades (30–39 yrs). As depicted in Table 3, on both sides. BMI and BSA strongly correlated with renal volume, particularly among the hypertensive patients.

**Table 3:** Pearson correlations between renal volume and somatic parameters in male and female hypertensive patients and controls^a^

		Hypertensive patients		Non-hypertensive controls	

Variable	Sex	Right RV	Left RV	Right RV	Left RV
Age	Female	0.132	0.083	-0.127	-0.011
	Male	-0.158	-0.179	-0.016	-0.194
	Both sexes	-0.011	-0.058	-0.075	0.057
Body weight	Female	0.538**	0.459**	0.381	0.328
	Male	0.583**	0.698**	0.364*	0.223
	Both sexes	0.531**	0.560**	0.366**	0.293*
Body height	Female	0.372*	0.231	0.267	0.145
	Male	0.547**	0.646**	0.391*	0.128
	Both sexes	0.472**	0.395**	0.353*	0.222
BMI	Female	0.463**	0.436**	0.288	0.295
	Male	0.367*	0.459**	0.150	0.187
	Both sexes	0.308*	0.383*	0.118	0.140
BSA	Female	0. 548**	0.456**	0.392*	0.307
	Male	0.620**	0.743**	0.423*	0.234
	Both sexes	0.576**	0.587**	0.425**	0.313*

a values are Pearson's correlation coefficients; BMI, body mass index; BSA, body surface area

** correlation is significant at the p < 0.01 level (2-tailed)

* correlation is significant at the p < 0.05 level (2-tailed)

In hypertensive patients, renal volume was correlated significantly (p<0.05) with BMI (r=0.308 and 0.383) for right and left kidneys, respectively. Further, significant positive correlation was also seen between renal volume and BSA in the hypertensive group =0.576 and 0.587 (p<0.01) for right and left kidneys respectively. When stratified by sex, these correlations were still strong and significant (Table 3). Among non-hypertensive controls, in contrast, only BSA showed significant correlation with renal volume on both sides in both sexes (Table 3).

## Discussion

In the recent past, the prevalence and absolute burden of the well-known modifiable risk factor of renal failure, hypertension (1), is rising globally especially in low- and middle-income countries, including Ethiopia (3,4,5). This study evaluated the impact of hypertension on the kidneys among hypertensive patients by undertaking ultrasonic renal measurements at Diagnostic Radiology department of Jimma University Medical Center (JUMC), Jimma, Ethiopia. For this purpose, abdominal ultrasound of both kidneys was performed for a sample of 145 adult Ethiopian populations (85 hypertensive and 60 healthy controls).

Accordingly, this study evaluated renal volume in patients with hypertension and healthy controls using ultrasound. The result shows that, the renal volume of the hypertensive group that ranged from 36.1 to 201.6 (mean=97.7) cm^3^ for the right kidney and 39.6 to 189.5 (mean=104.4) cm^3^ for the left kidney, was slightly smaller than the size calculated for nonhypertensive controls (RRV: between 61.8 and 159.5 (mean=101.1) cm^3^; LRV: range 35.8 to 253.7, mean=111.8 cm^3^). The renal volume obtained in the current population is comparable with results reported from Sudan among similar population (29), but smaller than that reported from Nigeria (20), possibly due to differences among ethnic and geographical differences. Ultrasonic renal size reports from Ethiopia are yet scarce (27).

Renal volume tends to appear in direct relationship with height, BMI and BSA in literature (20). In this study, we evaluated the relationships between renal volume and variables such as age, body weight, height, BMI and BSA, separately for hypertensive and nonhypertensive populations. The relationships obtained were in line with existing body of literature from Ethiopia and rest of the world (27,30,31). To mention few of these associations in our result, renal volume was positively and significantly correlated with BSA on both sides in both sexes among hypertensive patients, while the correlation was modest among nonhypertensive controls. Correlations between renal volume and BMI were also significant in both kidneys, as also reported in similar studies (29). We also revealed BSA has a better correlation with renal volume than BMI. Since the test of time in anatomical sciences, left kidney dominance over the right one with regard to their size is unquestionable and well described (32), which was also once again verified in the current study.

In clinical practice, bilateral renal shrink as a result of chronic disease, supports the diagnosis of CKD in long-standing disease duration (22). In our sample, we observed slightly smaller bilateral renal volume among hypertensive patients as compared their control counter parts. However, the difference was small and not significant. This finding is in agreement with a report from Turkey (16), which also reports reduced renal volume in hypertensive patients when compared with non-hypertensive controls.

Understanding a slight slide on a slippery slope could end with unprecedented deleterious sequelae with regard to renal failure, we adored to step further to insight if the observed minor shrinkage on renal volume as compared to that of the controls is due to the hypertension itself or other confounders. As already established, an individual's kidney size is directly related to body height, weight, BMI and BSA. In our further analysis, we accounted this body physique variation among study subjects with regard to renal size. This was by considering relative renal volume, by computing individual level renal volume to BSA ratio (RV/BSA). Our analysis showed significant shrinkage of both kidneys among hypertensive patients as compared to the mean scores the control group. Although this conclusion needs validation through larger studies, our preliminary result shows renal volume to body size ration better detect potential anatomical changes associated with long lasting chronic diseases over time. Literature reports considering ultrasonic renal volume to body size relation are currently inexistent.

The small sample size was one of the limitations of our study. Further, while attempting to provide insights on the impact of hypertension on the kidneys in this study, the approach focused only on anatomical aspects .i.e. ultrasonic renal size, regardless of pathophysiologic considerations of the kidneys.

## Conclusions

In our study, we provided measures of renal volume in patients with essential hypertension in southwest Ethiopia; the renal size was slightly smaller among hypertensive patients as compared to their control counter parts. The renal volume shows a significant positive correlation with body height, BMI and BSA.Our preliminary result also shows renal volume to body size ratio better probably detect potential anatomical changes associated with hypertension. Finally, we recommend large scale research on the rest regions of Ethiopia so that we will have fully standardized data on the subject.
